# Spiro‐Ometad As A Promising Substrate In Biomedical Devices

**DOI:** 10.1002/open.202400002

**Published:** 2025-01-10

**Authors:** Lisa Titze, Francesca Cadamuro, Nicoletta Murenu, Raziyeh Akbari, Luca Pellegrino, Giancarlo Capitani, Maurizio Acciarri, Carlo Antonini, Laura Russo, Norberto Manfredi

**Affiliations:** ^1^ Department of Materials Science and Milano-Bicocca Solar Energy Research Center – MIB-Solar University of Milano-Bicocca Via Cozzi 55 Milano I-20125 Italy; ^2^ School of Medicine and Surgery University of Milano-Bicocca Via Raoul Follereau 3 Vedano al Lambro (MB) I-20854 Italy; ^3^ Department of Earth and Environmental Sciences University of Milano-Bicocca Piazza della Scienza 1 Milano I-20126 Italy

**Keywords:** Spiro-OMeTAD, Collagen, Conductivity, Biosensors, Cytotoxicity

## Abstract

Bioactive films composed of Spiro‐OMeTAD, a conductive molecular material (CMM), in combination with collagen have been manufactured and characterised for the first time. In‐vitro cellular testing demonstrated the non‐cytotoxicity of the doped Spiro‐OMeTAD /Collagen films, opening the way for implantable or wearable medical devices and biosensors based on molecular materials.

## Graphical abstract

Spiro‐OMeTAD has been used for the first time to prepare a conductive biopolymer with collagen. Stability and electronic properties have been investigated. In‐vitro cellular testing showed the non‐cytotoxicity of films, paving the way for new medical devices and biosensors.

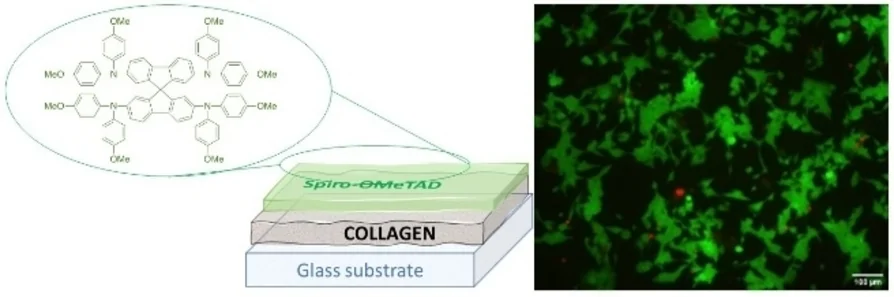

## Introduction

Developing conductive biomaterials is one of the key ingredients for developing multifunctional devices for tissue engineering and, in general, for biomedical applications. The use of conductive polymers embedded or conjugated to natural biopolymers, such as collagen or gelatine, is increasingly studied and characterized in the literature.^[1]^ However, conductive polymers suffer from limited biocompatibility due to the lack of molecular fragments for biorecognition, the combination with biopolymers is thus required to improve the integration with biological systems. Collagen is still one of the most widely employed polymers for the development of mesh, scaffolds or injectable biomaterials and medical devices. In the field of conductive polymers, the most widely used systems are the traditional materials based on conjugated anilines, thiophenes or thiophene derivatives, and pyrroles.^[2]^ Nonetheless, most of these materials come from the fields of photovoltaics^[3]^ and more in general from organic electronics^[4]^ and have been widely investigated in the last decades. However, to give them proper chemical and electrochemical properties suitable for bioelectronic applications,^2^, ^5^ doping^[6]^ and specific molecular side functionalization of these polymers have been extensively investigated to improve their performance and their compatibility with the biological environment.^[7]^ As a possible alternative strategy, molecular materials with defined structures, capable of conducting charges, either holes or electrons, have been developed and applied mainly in the field of solar cells.^[8]^ Moreover, molecular synthesis enables functionalization to modulate their optical and electronic properties and conjugate them, valorising conductive properties and tuning their wettability, so that, the possible interaction with the biological environment. Therefore, such molecular systems have a strong potential for applications in the biomedical field.

**Figure 1:**
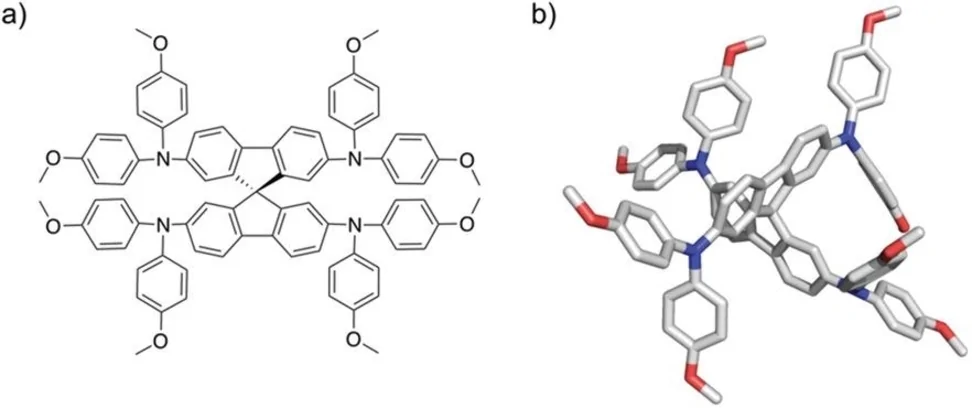
a) Chemical structure of Spiro‐OMeTAD and b) its crystal structure. Reprinted from ref.9b with permission. Copyright 2021 Wiley‐VCH GmbH.

This study aims to probe and demonstrate the potential molecular conductive material (CMM) for the fabrication of conductive and biocompatible biomaterials. For this first attempt a simple and effective molecule has been used, namely 2,2′,7,7′‐tetrakis(N,N‐bis(p‐methoxyphenyl)amino)‐9,9′‐spirobifluorene, (**Spiro‐OMeTAD**, see Figure 1),^[9]^ as CMM. **Spiro‐OMeTAD** has been chosen as it is a well‐known hole transport material, which is already widely used in organic photovoltaics applications.^[10]^ As a pristine material, **Spiro‐OMeTAD** is poorly conductive, with a conductivity of 10^−5^ mS/cm.^[11]^ Nonetheless, its conductive properties can be modulated by a variety of dopants, such as lithium bis(trifluoromethanesulfonyl)imide (LiTFSi),^[12]^ or by chemical oxidation with tris(2‐(1H‐pyrazol‐1‐yl)pyridine)cobalt(III) tri[bis(trifluoromethane)sulfonimide (FK102 Co(III) TFSI) reaching a conductivity of 10^−3^ S/cm.^11^, ^13^ Upon the above‐mentioned doping, this molecule showed good isotropic conductivity due to its amorphous nature making it a good candidate for the preparation of devices.^[14]^ Its synthesis is quite complex, as well as its functionalization, but the wide interest in this type of compound has pushed researchers to develop new cheap synthetic strategies for its preparation and functionalization.^[15]^

## Results and Discussion

The choice of **Spiro‐OMeTAD** lies in its widely investigated properties that are suitable for the purpose and its commercial availability. However, the doping procedure described above is incompatible with the biological environment, indeed, both cobalt and (trifluoromethane)sulfonimide salts should be avoided to improve biocompatibility. As such, in this study LiI replaces LiTFSI and FeCl_3_ is selected as an alternative to Co (III) salt as an oxidant.^[16]^

**Figure 2:**
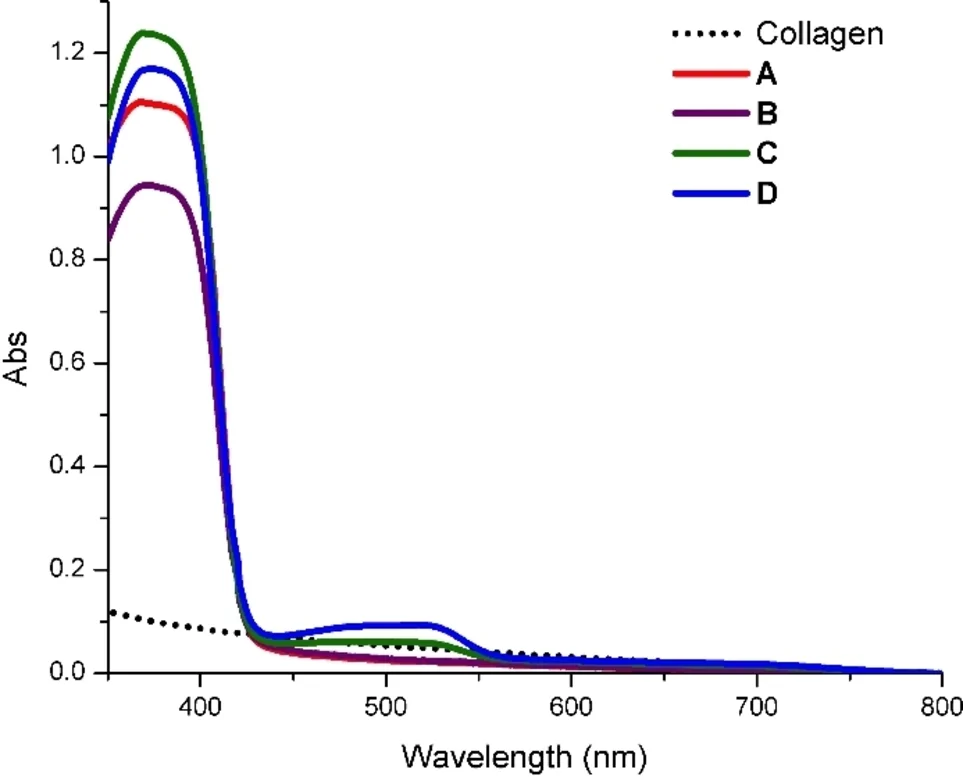
UV‐Vis spectra of collagen films functionalized with different formulations of CMM.

Both doped and undoped CMM were deposited on a collagen film over a glass substrate. Different formulations of CMM were tested to systematically verify the effect of each additive, based on our knowledge in photovoltaic application. Namely, four compositions of CMM, prepared according to the literature and reported in Table 1, were tested.^[17]^ The **A** sample was the pristine **Spiro‐OMeTAD**, **B** was Spiro‐OMeTAD doped with LiI, **C** was **Spiro‐OMeTAD** doped with FeCl_3_, and **D** sample was **Spiro‐OMeTAD** doped with both LiI and FeCl_3_. The so‐prepared films were investigated in their optical properties, their compatibility with the aqueous environment and their stability in biological media, both in acidic (pH 5.5) and alkaline condition (pH 7.4) to ensure their stability. Moreover, 48 h LIVE/DEAD tests on human glioblastoma cell line U87 were performed. First, collagen films were prepared by solvent casting,^[18]^ and second CMM was deposited using a spin‐coating technique on a 2×2 cm collagen‐glass substrate. The prepared CMM‐collagen materials **A, B, C**, and **D** were then characterised in their optical properties. The UV‐Vis spectra of the functionalized films are reported in Figure 2.

**Table 1:** Composition of different CMM solutions tested in the present work.

Sample	Spiro‐OMeTAD^[a]^ (mL)	LiI^[b]^ (μL)	FeCl3^[c]^ (μL)
**A**	1	–	–
**B**	1	17.5	–
**C**	1	–	29.7
**D**	1	17.5	29.7

^[a]^ 0.06 M in chlorobenzene. ^[b]^ 1.8 M in acetonitrile. ^[c]^ 0.2 M in acetonitrile.

UV‐Vis spectra showed the characteristic absorption band of the Spiro‐OMeTAD at 395 nm for all four samples (**A‐D)**. Furthermore, for both samples containing FeCl_3_(**C, D)**, **Spiro‐OMeTAD** oxidized species, in the form of a radical cation, caused a broad absorption band around 520 nm, in agreement with the literature.^[16]^ The intense absorption in the visible range enables an accurate evaluation of the undesirable material release during the stability tests.

Another relevant material property is the compatibility with aqueous environments. Indeed, correct cell adhesion on the material surface is an essential condition for *in vitro* or in case of *in vivo* applications, which is usually inadequate in conductive substrates. Surface wettability influences protein and cell adhesion. In detail, hydrophilic or partially hydrophilic surfaces – between 40° and 70° – have an improved ability to interact with different components of biological environments.^[19]^ Our materials interestingly possess the required wettability, as by contact angle (θ) analysis. Pure collagen is the most hydrophilic sample, with contact angles ranging from an initial 32° to 24° after three minutes. All four samples (**A**‐**D**) showed higher contact angles, with values in the range 50–70° after deposition, and 30–40° after three minutes. This trend can be explained by the hydrophobic nature of **Spiro‐OMeTAD** (θ=78°). Nonetheless, the contact angle is small enough to be compatible with cellular adhesion.^[20]^ Data are summarized in Table 2, and representative images are shown in Table S1. The values of θ were measured at different times on all samples where water drops showed a slow spreading after the initial deposition, likely due to partial adsorption of water in collagen.

**Table 2:** Contact angle measurements of collagen‐based functionalized films at different times. Values were measured as drops were deposited and after 3 minutes. The reported error refers to the standard deviation.

Sample	Contact angle θ (°)	
	t=0 (as deposited)	t=3 min
Collagen	33±2°	25±2°
Spiro‐OMeTAD	78°±1°	–
**A**	54°±1°	31°±2°
**B**	64°±10°	41°±5°
**C**	73°±7°	42°±5°
**D**	67°±2°	38°±2°

**Figure 3:**
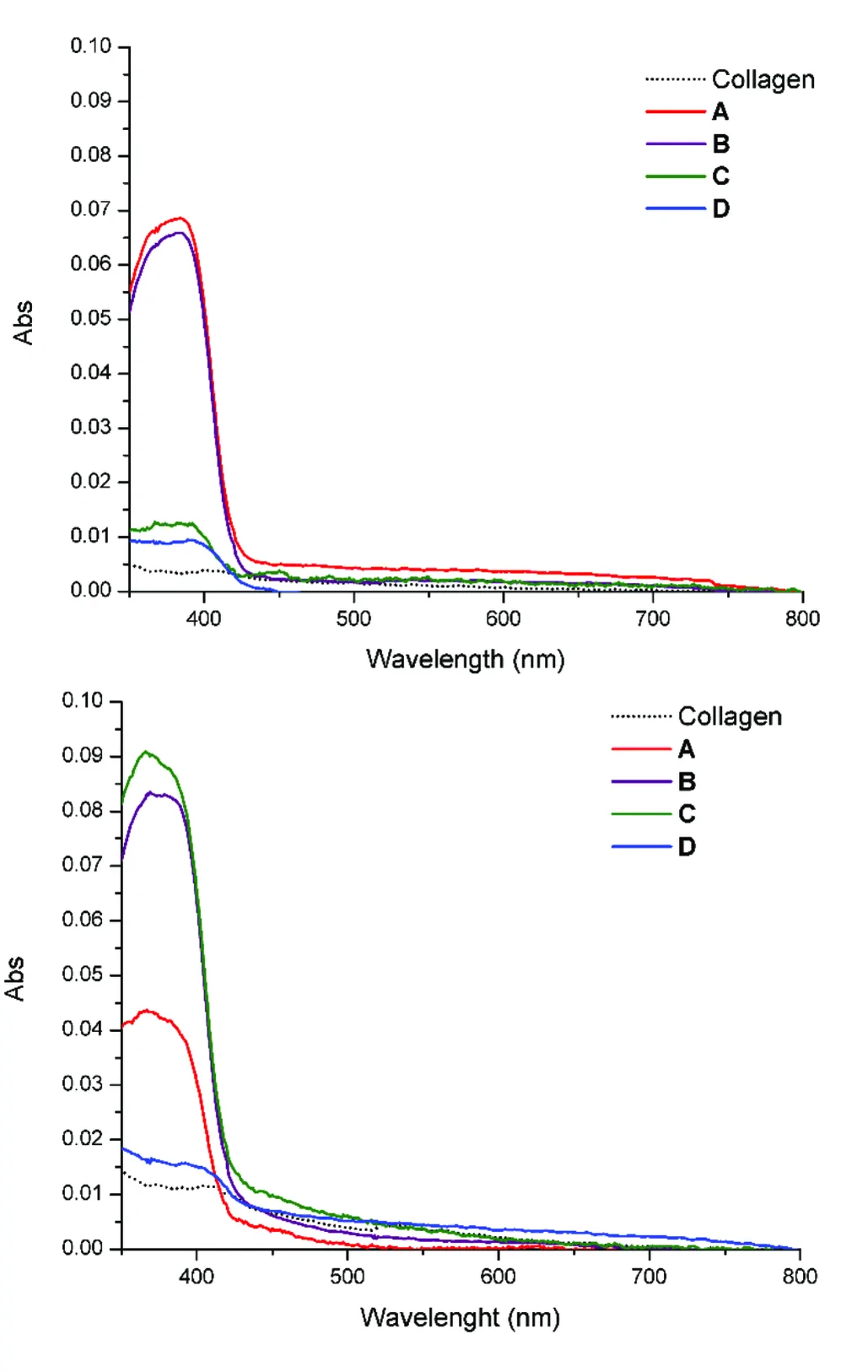
UV‐Vis spectra of stability test solution in (top) acidic and (bottom) alkaline PBS after 48 h.

The stability of the sample was then tested under physiological conditions and in mildly acidic conditions. A mildly acidic environment (pH 5.5) represents a pathological state, where the pH of cells and their surroundings is lower than the healthy condition (pH 7.4). The spectra of both films and solutions are shown in Figure 3. At pH 5.5 (Figure 3 top) samples **A** and **B** clearly show a release of the conductive material, as demonstrated by the absorption peak at 395 nm, corresponding to **Spiro‐OMeTAD**. For samples **C** and **D** negligible release is observed. This behaviour could be explained considering that samples **C** and **D**, in which **Spiro‐OMeTAD** is partially oxidized, can establish more intense electrostatic interactions with the collagen substrate on which they are deposited. The same analysis was conducted on alkaline solutions (Figure 3 bottom), showing that only sample **D** has no **Spiro‐OMeTAD** loss. Since that, sample **D**, with both LiI and FeCl_3_, performed best in the stability tests. So then, conductivity, morphology, and cytotoxicity were further investigated only on sample **D** and on pristine collagen as the control experiment.

A series of films of modified collagen (sample **D**) were tested in their conduction property by measuring their conductivity using van der Pauw's method as described in supporting information.^[21]^ The average value for conductivity is σ=1.5±0.3×10^−4^ S cm. This value is slightly lower than the best value reported in the literature and cited above for similar materials but is a good starting point for subsequent optimization.^[11]^ Moreover, the conductivity measurements allow us to verify the complete and continued coverage of the collagen surface.

**Figure 4:**
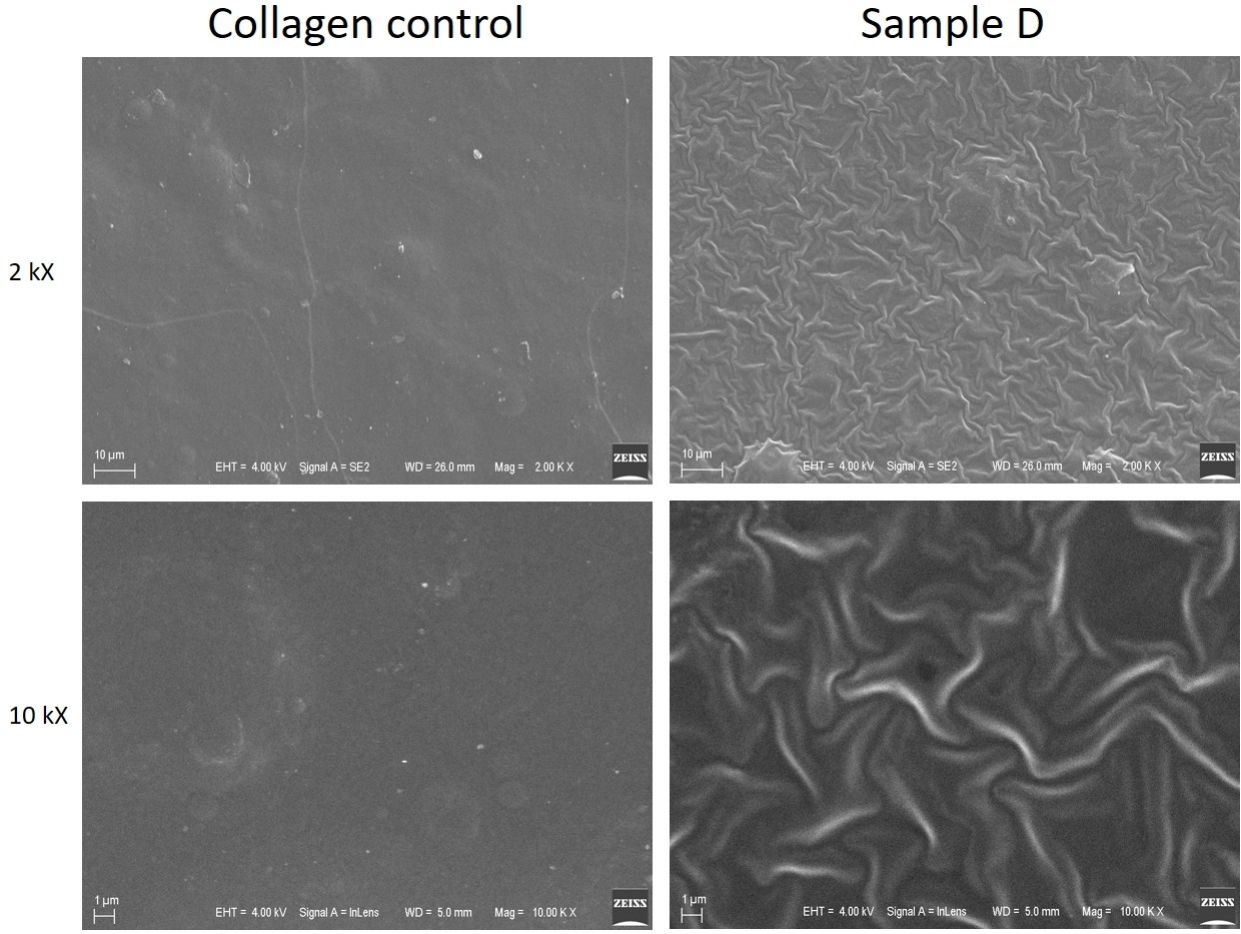
SEM images of collagen film (left) and Sample **D**‐collagen film (right) at 2 kX (upper) and 10 kX (lower) (for explanation see text).

SEM images of sample **D** and pure collagen films are reported in Figure 4 and Figure S3. The pure collagen control sample shows a smooth and continuous surface, altered only by small bulges and folds, possibly due to non‐uniform adhesion on the supporting glassy slide. Some (vertical and horizontal) bright, thin lines are observed at low magnification, possibly representing joints or terraces. Finally, some microscopic particles, perhaps dust deposited during the preparation, are also observed. Sample **D** has a completely different appearance. The **Spiro‐OMeTAD** coating is continuous but appears wrinkled and separated into microscopic, irregular islands. It is unclear if these islands formed during deposition or after exposure to the high vacuum of the SEM.

To further analyse the impact of the CMM over the macroscopic roughness, which is of major concern in terms of cell adhesion, 3D maps of the same spot of pristine collagen before and after the deposition of the CMM (sample **D)** were collected using a Stylus Profiler. The obtained images are depicted in Figure S4. The acquired data showed that the surface root‐mean‐square roughness parameter (Sq) in pristine collagen (1.049 μm) is greater than that of the functionalised counterpart (0.993 μm) demonstrating, at a macroscopic level, the different morphology of the two films.

**Figure 5:**
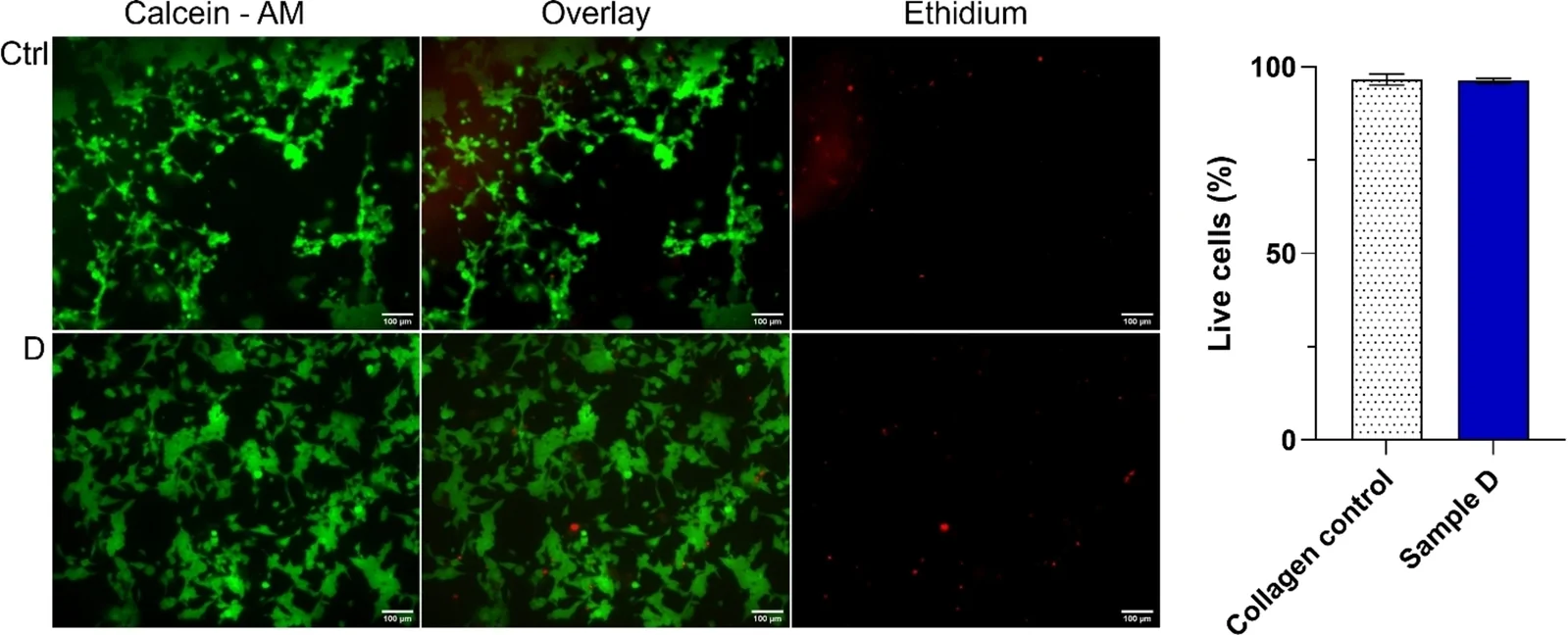
A) Live/Dead images of U87 cells on collagen control film and Sample **D** after 48 h (Scale bar 100 μm), B) percentage of viability in samples (mean ± SD).

To investigate the biocompatibility of Sample **D** we used U87 glioblastoma cells. U87 cells are optimal candidates to preliminary evaluate cytotoxicity for biomaterials and devices for nervous system applications, including in vitro advanced pathological models.^[22]^ Cell adhesion and cytotoxicity of sample **D**, was investigated with LIVE/DEAD assay and compared to the pristine collagen film as a control (Figure 5). The fluorescent images confirmed acceptable viability of the U87 cells after 48 h on both Sample **D** film and control collagen sample, showing a percentage of live cells over 96 % in both samples (Table S2). Therefore, the material is not cytotoxic and guarantees cell adhesion as in the control sample. Furthermore, even if a similar cell behaviour was observed in both the samples, cells cultured on Sample **D** are more able to adhere homogeneously on the entire surface, if compared to the cells cultured on collagen control film. This preliminary observation suggests an improved adhesion and population on the treated surface opening the way to further investigations and application for the development of engineered in vitro models.

## Conclusions

In conclusion, a new promising conductive biomaterial was prepared to exploit collagen films by solvent casting, on which **Spiro‐OMeTAD** doped with FeCl_3_ was deposited using a spin‐coating technique. The obtained materials showed satisfactory stability in both physiological and pathological pH. Furthermore, preliminary cytotoxicity and wettability tests indicate the biomaterial compatibility for in vitro and in vivo applications. These results therefore pave the way to conductive molecular materials as a potential alternative to traditional polymeric systems. conductive molecular material properties and their great structural variety enable the development of new hybrid materials obtained by conjugation with biopolymers. Thus, there is plenty of room to develop both fundamental and applied research in this field. We hope this preliminary work may contribute to popularising the use of CMMs in bioelectronics.

## Experimental Section

### Materials and Methods

Spiro‐OMeTAD, LiI and FeCl_3_ have been purchased from Sigma Aldrich and used without further purification. UV‐O_3_ treatment was performed using Novascan PSD Pro Series – Digital UV Ozone System. The thickness of the layers was measured by means of a VEECO Dektak 8 Stylus Profiler. For conductivity measurements, Keithley 617 which has a high internal input impedance (>200 TΩ) was used in combination with the Keithley 224 as the current source. Collagen from bovine Achilles tendon, live/dead Cell viability Assay kit (CBA415), Phosphate‐Buffered Saline, U87 glioblastoma cell line, Eagle's minimal essential medium, L‐glutamine, Sodium Pyruvate, Fetal Bovine Serum, penicillin, and streptomycin were purchased from Sigma‐Aldrich.

### Film Preparation

The collagen films were prepared by a solvent‐casting method as previously described.^18^, ^23^ Briefly, 690 mg of Collagen from bovine Achilles tendon (Sigma – Aldrich) were dissolved in 315 mL AcOH water solution 0.5 M. The solution was stirred at 40 °C for four hours and then homogenized with a mixer for 2 min at maximum speed. 40 mL of final mixture was poured into an 8.5×12.5 cm^2^ plastic multiwell lid and and left under the hood until the solvent was completely evaporated. The films were washed three times with distilled water and then one time with ethanol. The wet films were dried under the hood and then stored at room temperature. The samples were cutted in pieces (1×1 cm) and hydrated with distillated water to adhere on a flat glass substrate (1×1 cm) and left under the hood until the solvent was completely evaporated.

### CMM Deposition

The glass substrates prepared as described above were treated in an ozone cleaner for 20 min before the CMM blend (100 μL/cm^2^), with the composition described in the main text Table 1, was deposited via spin coating on the glass‐coated substrate described above at 3000 rpm for 30 s in a nitrogen‐filled glove box.

### UV‐Vis Measurements

Absorption spectra were recorded with a V‐570 Jasco spectrophotometer. For investigation of the optical properties of the functionalized collagen, the films were deposited on a glass slide. For stability tests, the samples were immersed in PBS solution at pH 5.5 and 7.4 for 48 hours, then the films were collected and analysed by UV‐Vis. The solutions were freeze‐dried, and the precipitate salts and any eventually released film particles were analysed. The obtained solid was dissolved in DMSO and filtered to remove inorganic salt traces, and the presence of CMMs in the solution was determined by UV‐Vis analysis

### Contact Angle Measurements

The contact angle analysis is performed using an in‐house contact angle setup, consisting of a camera (Fastcam Nova S6, Photron) with Tokina AT−X PRO D (100 mm F2.8 MACRO) as the optical lens and backlight illumination. Deionized water is dispensed to form a drop on the surface (volumes in the range of 5–10 μl) using a syringe pump (Harvard Apparatus, Pump 11 Pico Plus Elite). Contact angles (θ) are measured upon deposition and after 3 minutes for pristine collagen and all four samples (A−D), as well as for pure Spiro‐OMeTAD on glass, as a reference. Table S1 includes representative images, from which contact angle values are extracted. Please refer to Table 2 in the manuscript for contact angle values and statistics.

### Conductivity Measurements

The conductivity (σ) value is obtained from the resistivity as σ=1/ρ. We used the van der Pauw technique for the resistivity (ρ) measurements at room temperature.^[21]^ For a more detailed description of the method see the dedicated section in supplementary materials.

### Scanning Electron Microscopy (SEM)

Sample **D** and pristine collagen films were placed on glass slides and coated with a 20 nm carbon film, then analysed by Scanning Electron Microscopy at the Platform of Microscopy of Milano‐Bicocca using a ZEISS Gemini 500 field emission SEM. Images were taken at a voltage of 4 kV, from 500X to 20kX, using secondary electrons.

### Roughness Evaluation

The roughness of the surfaces was performed using a Dektak 8 Stylus Profiler (Veeco Instruments, Inc.) equipped with an N‐Lite low force sensor with a computerised surface and a high sensitivity was used to measure the 3D maps with an accuracy in the range of 0.20 microns. The samples were characterised using a 2.5‐μm‐radius tip with a recording speed of 0.150 mm/s and a loading force of 0.03 mg. Map images were acquired with a Y resolution of 12.7 μm. Data were analysed using Vision64 software by Bruker. The Sq parameter was calculated as by the following formula:

$$Sq=\sqrt{\frac{1}{A}\;\int \int \;Z^{2}\left(x,y\right)dxdy}$$

where A is the surface area and Z is the relative height of the peak.

### Cell Culture

Human glioma U87‐MG cells were cultured in Dulbecco's modified Eagle's medium supplemented with 10 % fetal bovine serum, 100 units/ml penicillin, and 100 mg/ml streptomycin. Cells were grown to 90 % confluence and then trypsinized, plated in 75 cm^2^ culture dishes at a density of 1.5×10^6^ cells and incubated at 37 °C in a humidified atmosphere containing 5 % CO_2_. The medium was changed every three days.

### Live/Dead Assay

Sample **D** (1×1 cm) was gently detached from the glass slide by hydrating it with distilled water and placed in a 24‐well plate with the functionalized side facing upward. Ethanol was used to help spread the collagen film. The sample and a pristine collagen film (1×1 cm), used as a control, were then hydrated for 1 hour with medium in the 24‐well plate. U87 10^5^ cells/well were loaded on top of each film. Three replicates for each sample were analysed to ensure reproducibility. After 48 h, U87 were stained with red‐fluorescent ethidium homodimer‐1 to detect the loss of plasma membrane integrity, and green fluorescent calcein‐AM to detect intracellular esterase activity for 20 min (37 °C, 5 % CO_2_) (Live/Dead Cell viability Assay kit (CBA415). Fluorescence images were collected with CELENA®S, Logos Biosystems. Cell viability was calculated as (number of green‐stained cells/number of total cells)×100).^[24]^

## Conflict of Interests

The authors declare no conflict of interest.

## Supporting information

As a service to our authors and readers, this journal provides supporting information supplied by the authors. Such materials are peer reviewed and may be re‐organized for online delivery, but are not copy‐edited or typeset. Technical support issues arising from supporting information (other than missing files) should be addressed to the authors.

Supporting Information

## Data Availability

The data that support the findings of this study are available from the corresponding author upon reasonable request.
