# Supplementary material for: Spiro‐Ometad As A Promising Substrate In Biomedical Devices
Source: ChemistryOpen. 2025 Jan 10;14(5):e202400002. doi: 10.1002/open.202400002 (PMC13580777; doi:10.1002/open.202400002)
Supplement: Supplementary file 1 [file OPEN-14-e202400002-s001.pdf]

ChemistryOpen

Supporting Information

Spiro-Ometad As A Promising Substrate In Biomedical Devices

Lisa Titze, Francesca Cadamuro, Nicoletta Murenu, Raziyeh Akbari, Luca Pellegrino, Giancarlo Capitani, Maurizio Acciarri, Carlo Antonini, Laura Russo,* and Norberto Manfredi*

Supporting information

Table S1 Images of contact angle measurements at different times. 2

Conductivity measurements method and sample preparation..2

Figure S1 Sample geometry used conductivity measurements. 3

Figure S2 Schematic configurations for van der Pauw's resistivity measurement. 3

SEM Images 4

Figure S3 SEM images of collagen film (left) and Sample D-collagen film (right) at 500 X (upper) and 20 kX (lower) (for explanation see manuscript text). 4

Figure S4 2D (top) and 3D (bottom) surface maps of collagen film (left) and Sample D-collagen film (right)..5

Biological experiments analysis 5

Table S2: Numeric Data from Live/Dead assay quantification 5

Bibliography 5

Table S1 Images of contact angle measurements at different times.

Sample	Sessile drop images for contact angle measurement	
	t = 0 (as deposited)	t = 3 min
Spiro-OMeTAD	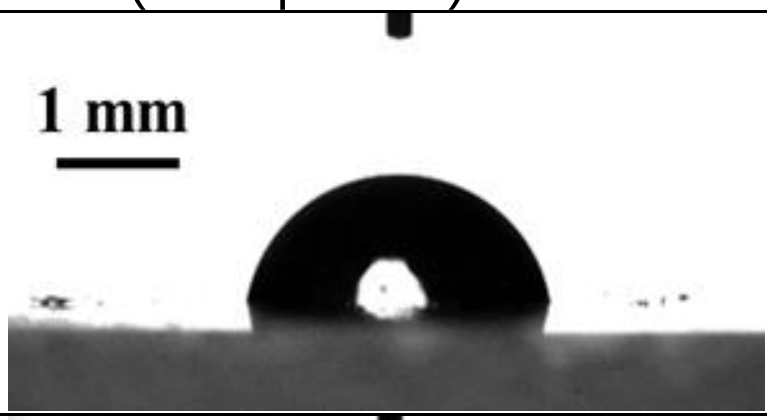	-
A	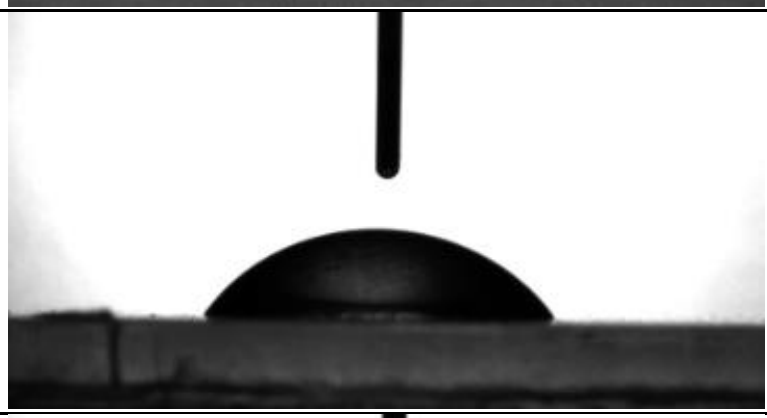	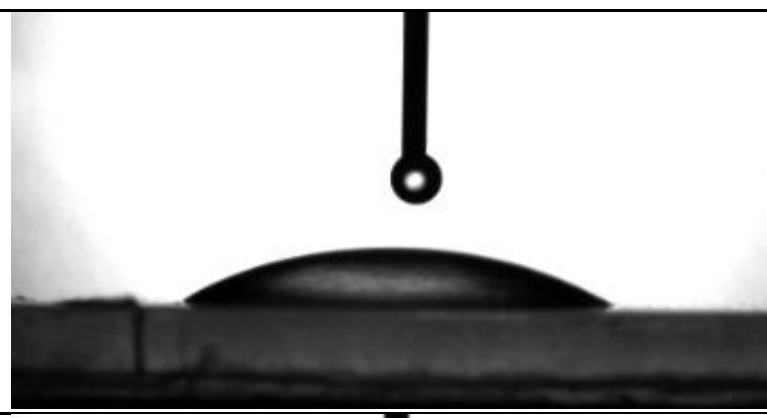
B	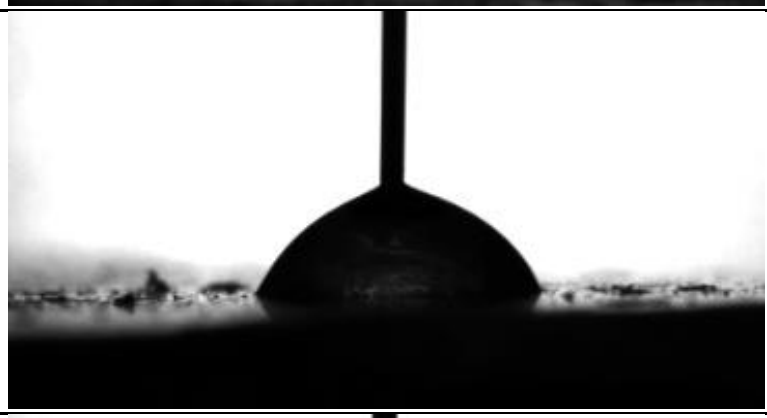	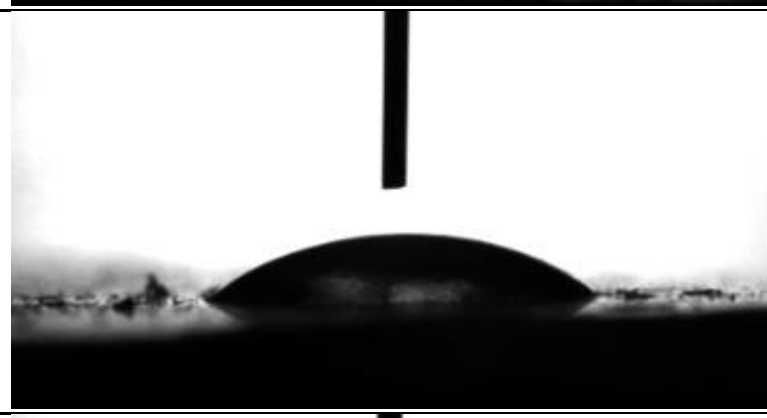
C	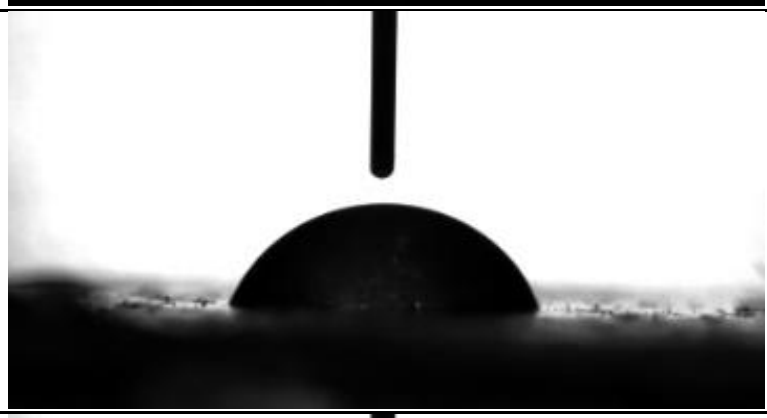	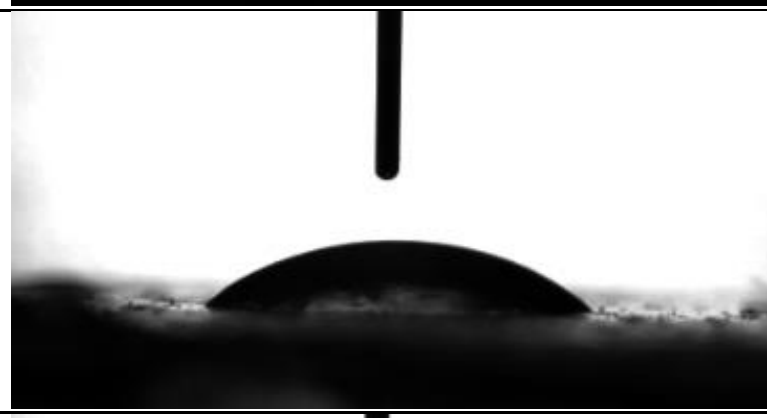
D	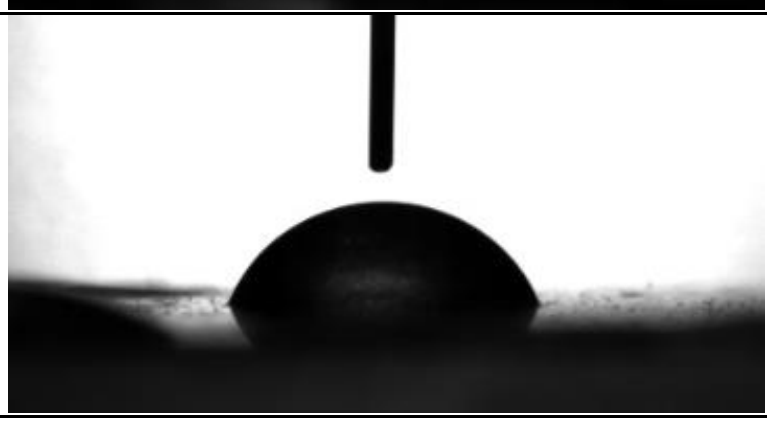	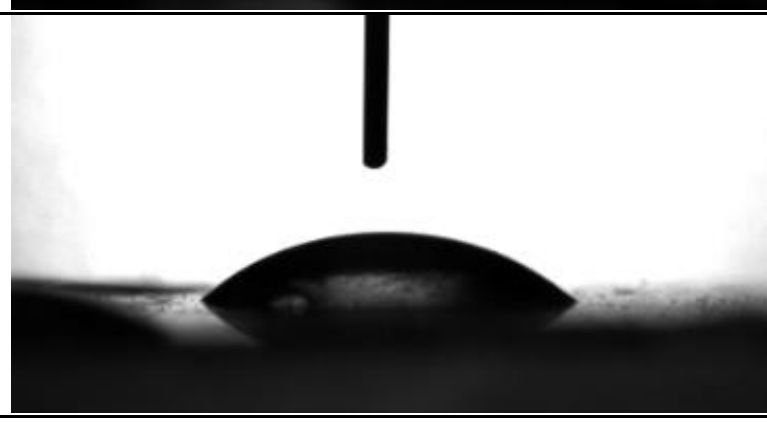

Conductivity measurements method and sample preparation

The conductivity (σ) value is obtained from the resistivity as $\sigma=1/\rho$. We used the van der Pauw technique for the resistivity (ρ) measurements at room temperature.[1] The van der Pauw technique requires only four contacts located anywhere on the periphery of a uniformly thick sample of arbitrary shape. The samples, prepared as described above, were first cut into 1 x 1 cm squares after which we deposited small dots of either silver (by evaporation) or gold (by sputtering) at each corner of the samples (Figure S1). The samples were not annealed after the metal deposition. We did not find significant differences in the results between the two metals used.

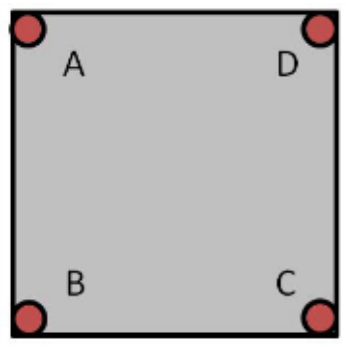

Figure S1 Sample geometry used conductivity measurements.

With a van der Pauw system, it is necessary to measure across different pairs of contacts to correct for the geometry, which requires interchanging current and voltage leads to the sample as shown schematically for a square specimen in Figure S2.

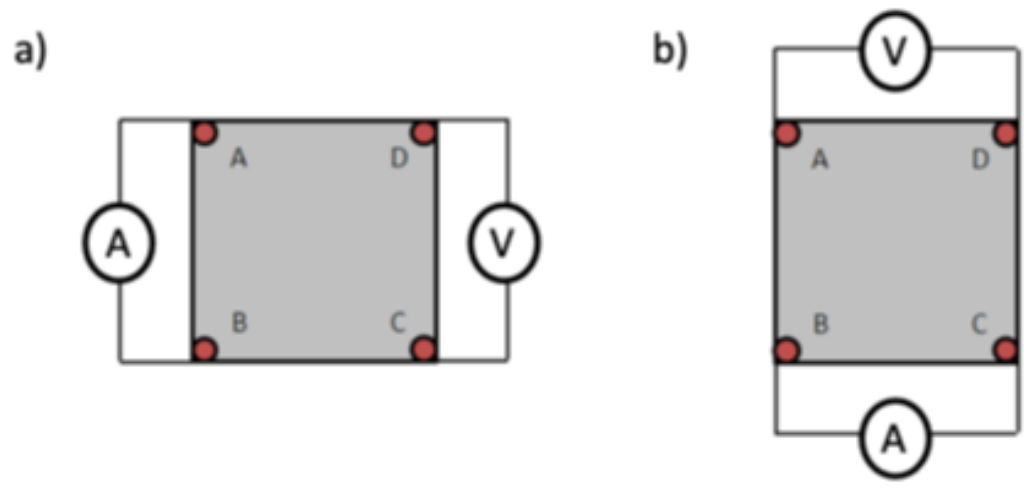

Figure S2 Schematic configurations for van der Pauw's resistivity measurement.

For the van der Pauw method, we define the equivalent resistance $R_{AB;CD}$ as one as the relationship between the potential difference (*V*) between contacts D and C and the current (*i*) flowing between A and B (Figure 2a):

$$R_{AB;CD} = \frac{V_{cd}}{i_{AB}}$$

We then measured also the equivalent resistance $R_{BC;DA}$ where it is the relationship between the potential difference between contacts D and A and the current flowing between B and C (Figure 2b):

$$R_{BA;DA} = \frac{V_{cd}}{i_{AB}}$$

The resistivity, in ohms-centimetre, is calculated using Eq. (1)

$$\rho = \frac{\pi d}{ln2}\left(\frac{R_{AB;CD}+R_{AB;CD}}{2}\right) f\left(\frac{R_{AB;CD}}{R_{AB;CD}}\right) \qquad [1]$$

Where $f(\frac{R_{AB;CD}}{R_{AB;CD}})$ is a dimensionless quantity dependent only upon the ratio of $\frac{R_{AB;CD}}{R_{AB;CD}}$ that in our case was approximated at 1 thanks to the quite good square shape and symmetry of our samples. A sample thickness of about d = 200 ± 50 nm was measured by profilometer.

From the preliminary measurements carried out, we realised our samples have high resistivity. For this reason, we used for the voltage measurements the electrometer Keithley 617 which have a high internal input impedance (>200 TΩ). As the current source, the Keithley 224 was used. The current used was in the range of 1-5 x 10^{-8} A and the voltage measured in the range of 0.5-4 Volt. We repeated the measurements 3 times for each configuration at currents and the different metals used.

SEM Images

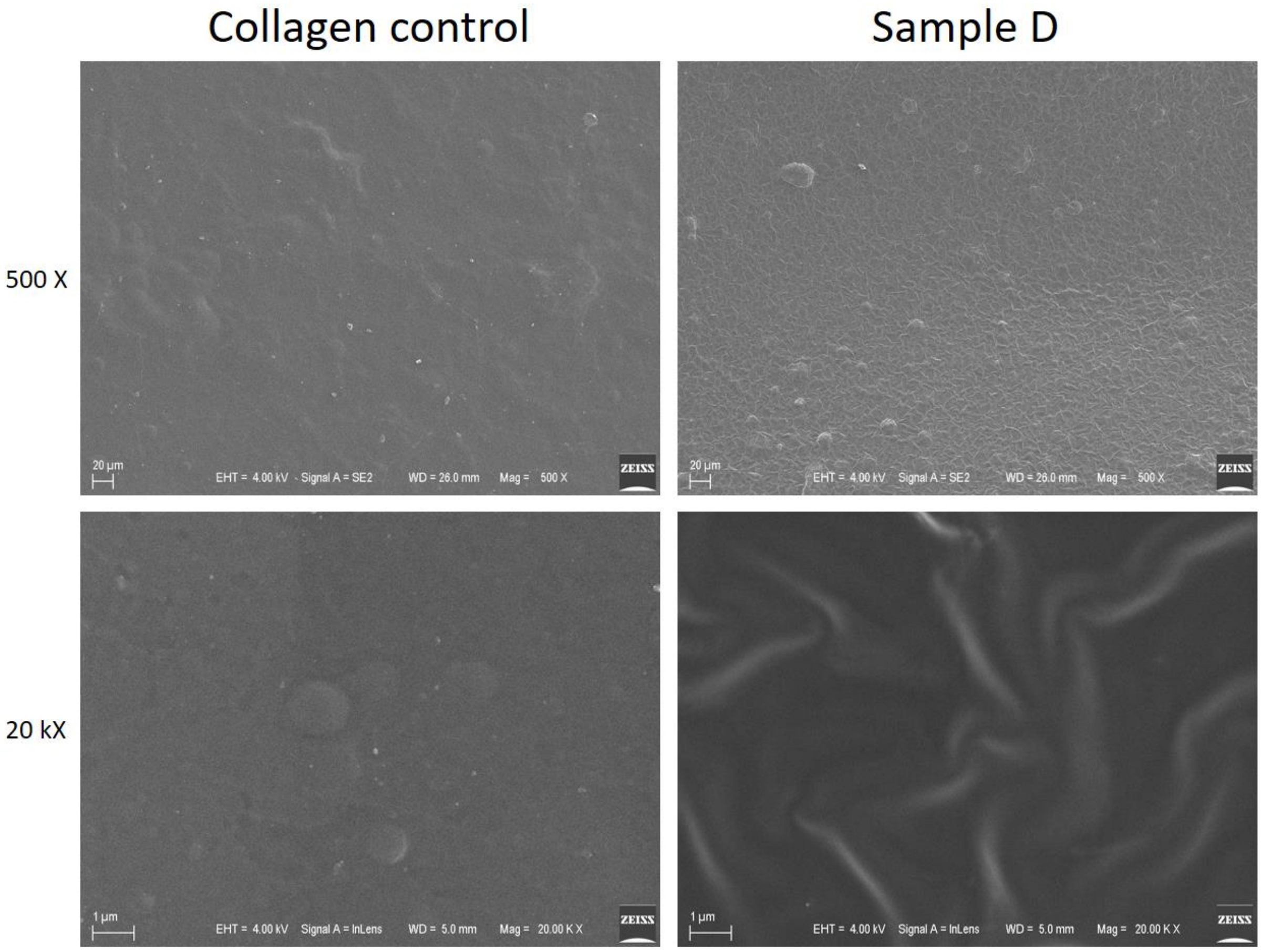

Figure S3 SEM images of collagen film (left) and Sample D-collagen film (right) at 500 X (upper) and 20 kX (lower) (for explanation see manuscript text).

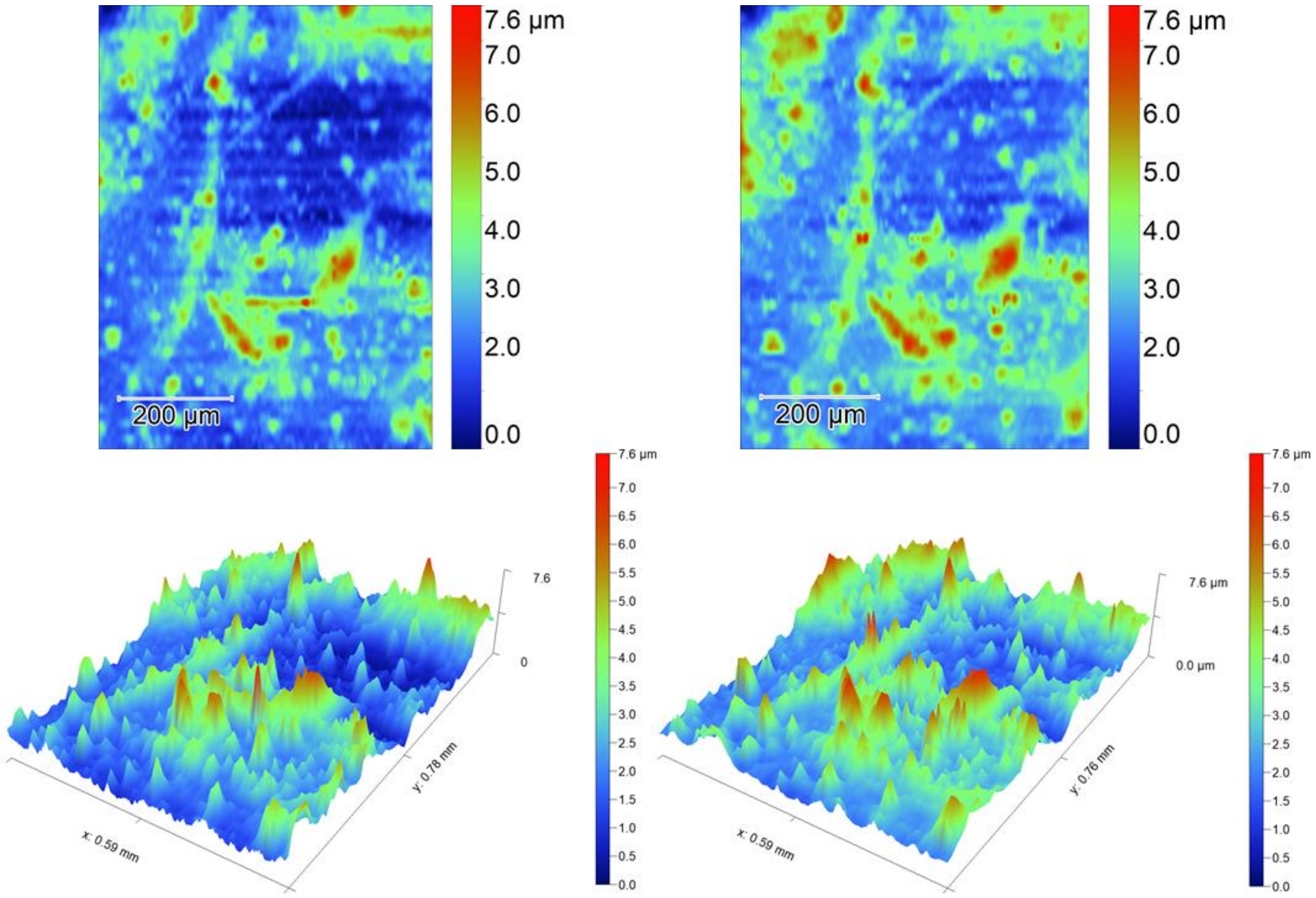

Figure S4 2D (top) and 3D (bottom) surface maps acquired on the same spot, before (left) and after (right) CMM deposition, to evaluate the effect on surface roughness**.**

Biological experiments analysis

Table S2: Numeric Data from Live/Dead assay quantification

	Sample	Live	Tot Cells	% Viability	Viability Average %	SD
CTRL	C1	158	165	95,8	96,7	1,56
	C2	127	129	98,4		
	C3	90	94	95,7		
Sample D	A1	126	130	96,9	96,3	0,64
	A2	154	161	95,7		
	A3	126	131	96,2		

Bibliography

[1] L. J. van der Pauw, *Philips Res. Rep.*, **1958**, *13*, 1-9.
